# Publisher Correction: The SecM arrest peptide traps a pre-peptide bond formation state of the ribosome

**DOI:** 10.1038/s41467-024-47509-9

**Published:** 2024-04-16

**Authors:** Felix Gersteuer, Martino Morici, Sara Gabrielli, Keigo Fujiwara, Haaris A. Safdari, Helge Paternoga, Lars V. Bock, Shinobu Chiba, Daniel N. Wilson

**Affiliations:** 1 Institute for Biochemistry and Molecular Biology, University of Hamburg, Martin-Luther-King-Platz 6, 20146 Hamburg, Germany; 2 Theoretical and Computational Biophysics Department, Max Planck Institute for Multidisciplinary Sciences, Göttingen, Germany; 3 Faculty of Life Sciences and Institute for Protein Dynamics, Kyoto Sangyo University, Kamigamo, Motoyama, Kita-ku, Kyoto 603-8555 Japan

**Keywords:** Ribosome, Cryoelectron microscopy

Correction to: *Nature Communications* 10.1038/s41467-024-46762-2, published online 19 March 2024

The original version of this Article contained an error in ref. 74, which was incorrectly given without a publication date. The correct form of ref. 74 is: Morici, M., Gabrielli, S., Fujiwara, K. et al. RAPP-containing arrest peptides induce translational stalling by short-circuiting the ribosomal peptidyltransferase activity. *Nat Commun* **15**, 2432 (2024). This has been corrected in the PDF and HTML versions of the Article.

