# Supplementary material for: The SecM arrest peptide traps a pre-peptide bond formation state of the ribosome
Source: Nat Commun. 2024 Mar 19;15:2431. doi: 10.1038/s41467-024-46762-2 (PMC10951299; doi:10.1038/s41467-024-46762-2)
Supplement: Supplementary file 1 — Supplementary Information [file 41467_2024_46762_MOESM1_ESM.pdf]

Supplementary Information

The SecM arrest peptide traps a pre-peptide bond formation state of the ribosome

Felix Gersteuer[1,#], Martino Morici[1,#], Sara Gabrielli[2], Keigo Fujiwara[3], Haaris A. Safdari[1], Helge Paternoga[1], Lars V. Bock[2], Shinobu Chiba[3], Daniel N. Wilson[1,*]

[1] Institute for Biochemistry and Molecular Biology, University of Hamburg, Martin-Luther-King-Platz 6, 20146 Hamburg, Germany.

[2] Theoretical and Computational Biophysics Department, Max Planck Institute for Multidisciplinary Sciences, Göttingen, Germany

[3] Faculty of Life Sciences, Kyoto Sangyo University, Kamigamo, Motoyama, Kita-ku, Kyoto 603-8555, Japan.

[#] These authors contributed equally

*Correspondence to:

Daniel Wilson (Daniel.Wilson@chemie.uni-hamburg.de)

Contents

Supplementary Figures 1-12

Supplementary Tables 1-4

Supplementary References

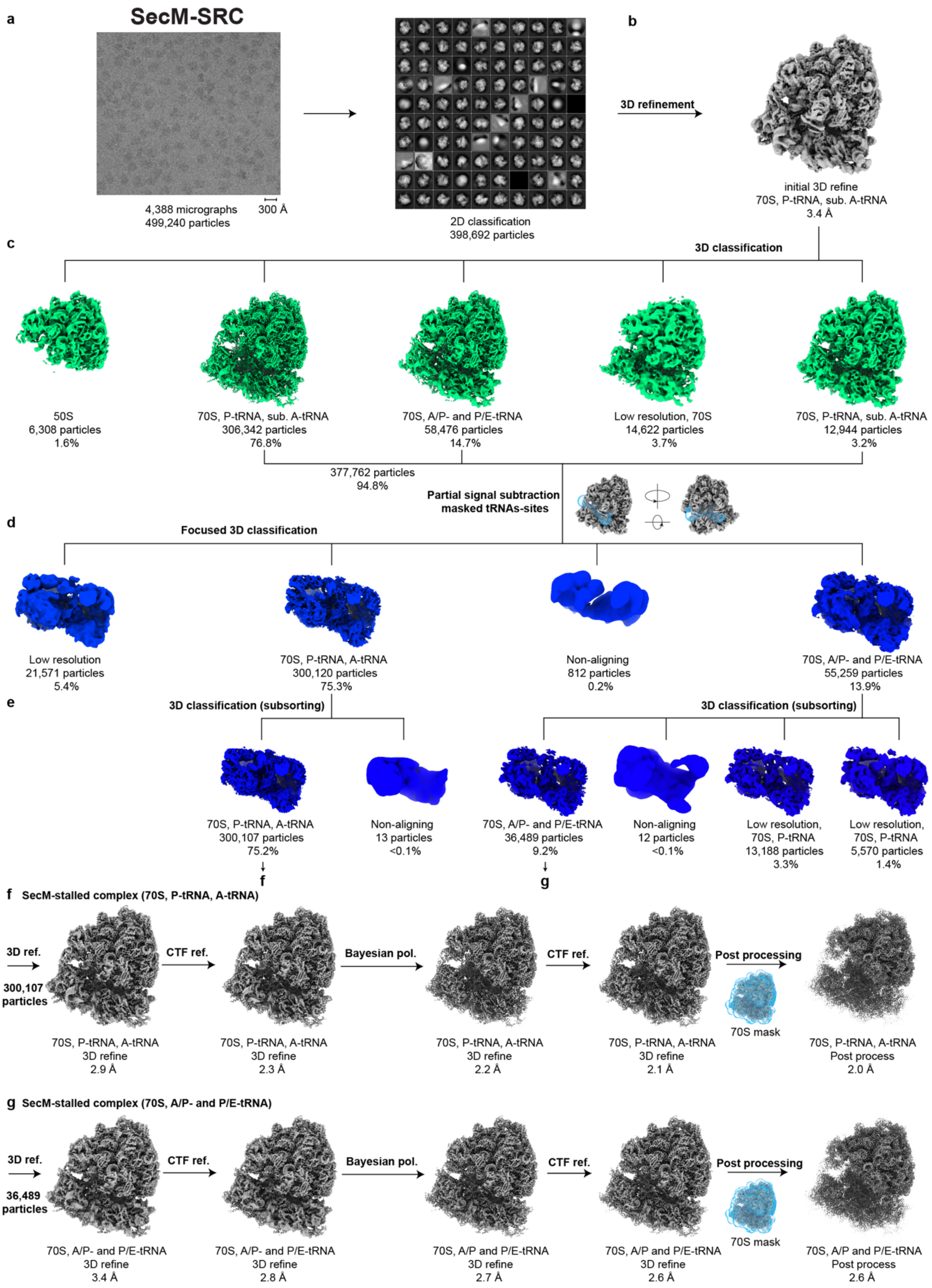

Supplementary Figure 1 ***In silico*** **sorting scheme for SecM.** (**a**) From 4,388 micrographs, 499,240 particles were picked and subjected to 2D classification resulting in 398,692 ribosome-like particles. Particles were (**b**) initially 3D-refined, then (**c**) subsorted into five classes. 70S-like particles were pooled and (**d**) subsorted into four classes using a mask around the tRNA binding sites. Two major classes with both A- and P-tRNA density (75.3%) and hybrid A/P- and P/E-tRNA density (13,9%) were further (**e**) subsorted into 2 and 4 classes, respectively. The major classes of this subsorting with (**f**) both A- and P-tRNA density (75.2%) and (**g**) hybrid A/P- and P/E-tRNA density (9.1%) were processed further, resulting in final average resolutions (at FSC 0.143) of 2.0 Å and 2.6 Å, respectively.

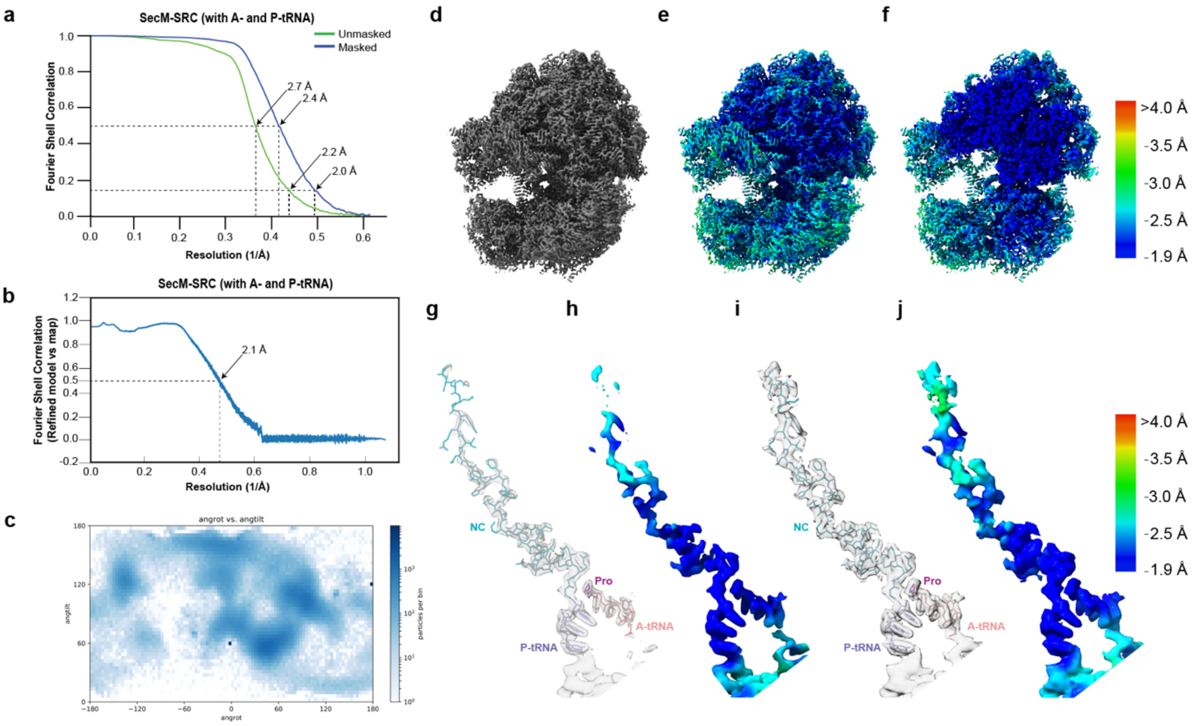

Supplementary Figure 2 FSC and local resolution for SecM-SRC. (**a**) Fourier shell correlation (FSC) curve of the SecM-SRC containing A- and P-tRNA, with unmasked (green) and masked (blue) FSC curves plotted against the resolution (1/Å). (**b**) Refined model vs map FSC curve of the SecM-SRC containing A- and P-tRNA plotted against the resolution (1/Å). (**c**) Angular distribution of particles used for 3D reconstruction from Relion. Particles are binned and logarithmical represented from white to blue. (**d-f**) Cryo-EM density for the 3D-refined map of the SecM-SRC, coloured (d) grey, and (e-f) according to local resolution. In (f), a transverse section reveals the core of the 50S subunit, including the ribosomal exit tunnel. (**g-h**) Cryo-EM density for the peptidyl-tRNA in the P-site and Pro-tRNA in the A-site of the 3D-refined map of the SecM-SRC, coloured in grey (g) and according to local resolution (h). (**i-j**) Same representation as in (g-h) but at a lower threshold to reveal density in the N-terminal region of the nascent chain.

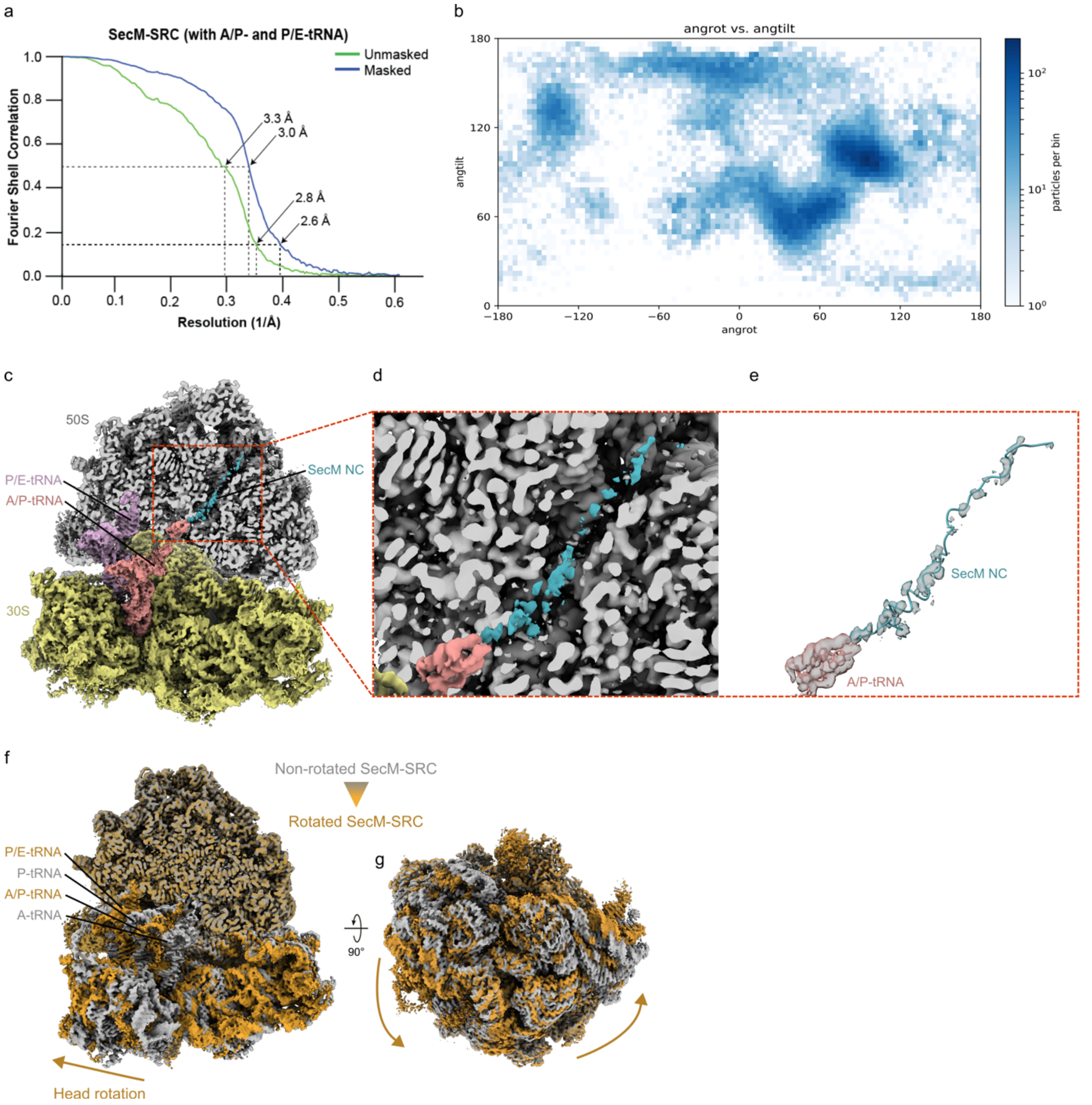

Supplementary Figure 3 Minor SecM-SRC complex with hybrid A/P- and P/E-tRNA densities. (**a**) Fourier shell correlation (FSC) curve of the SecM-SRC containing hybrid A/P- and P/E-tRNA, with unmasked (green) and masked (blue) FSC curves plotted against the resolution (1/Å). (**b**) Angular distribution of particles used for 3D reconstruction from Relion. Particles are binned and logarithmical represented from white to blue. (**c**) Cryo-EM map of the 3D-refined *E. coli* SecM-SRC with hybrid A/P- and P/E-tRNAs and transverse section of the 50S (grey) to reveal density for the fragmented nascent chain (teal), A/P-tRNA (salmon), P/E-tRNA (lavender) and 30S (yellow). (**d**) Zoom on the fragmented nascent chain (teal) in the minor SecM-SRC complex with transverse section of 50S (grey). (**e**) Extraction of the fragmented nascent chain density with SecM (teal) and P-tRNA from the major SecM-SRC complex containing A- and P-tRNA fit. (**f-g**) Overview of non-rotated major SecM-SRC complex (grey) vs rotated minor SecM-SRC complex (orange).

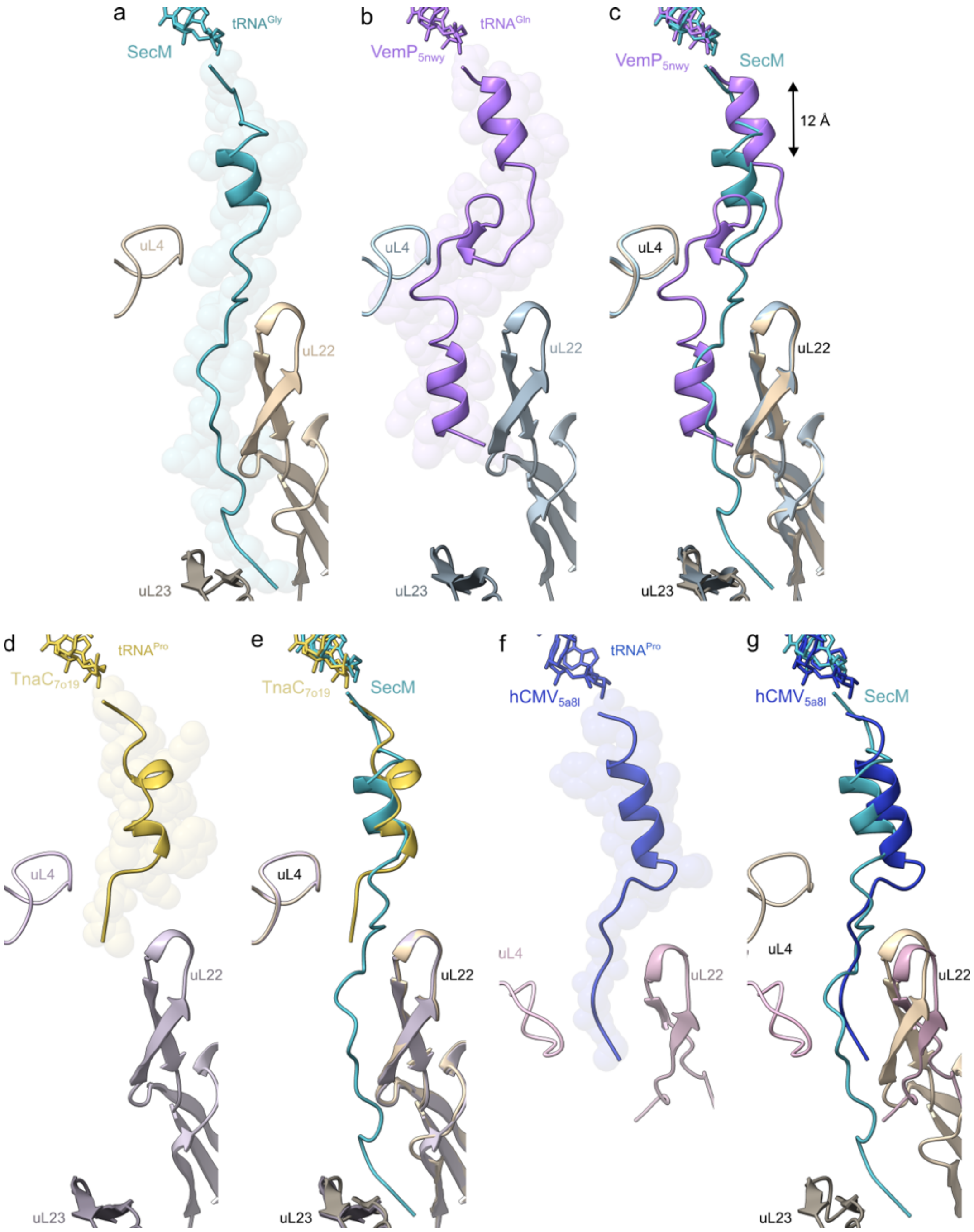

Supplementary Figure 4 Comparison of SecM with VemP, TnaC and hCMV arrest peptides. (**a**) SecM attached to the P-tRNA (teal) in relation to uL4 (light gold), uL22 (gold) and uL23 (dark gold). (**b**) VemP (PDB ID 5NWY)[1] attached to the P-tRNA (purple) in relation to uL4 (light slate blue), uL22 (slate blue) and uL23 (dark slate blue). (**c**) Overlay (aligned on the basis of 23S rRNA) of (b) VemP and (a) SecM (coloured as in a). (**d**) TnaC (PDB ID 7O19)[2] attached to the P-tRNA (yellow) in relation to uL4 (light grey rose), uL22 (grey rose) and uL23 (dark grey rose). (**e**) Overlay (aligned on the basis of 23S rRNA) of (d) TnaC and (a) SecM (coloured as in a). (**f**) hCMV (PDB ID 5A8L)[3] attached to the P-tRNA (blue) in relation to uL4 (light pink) and uL22 (pink). (**g**) Overlay of (f) hCMV and (a) SecM (coloured as in a).

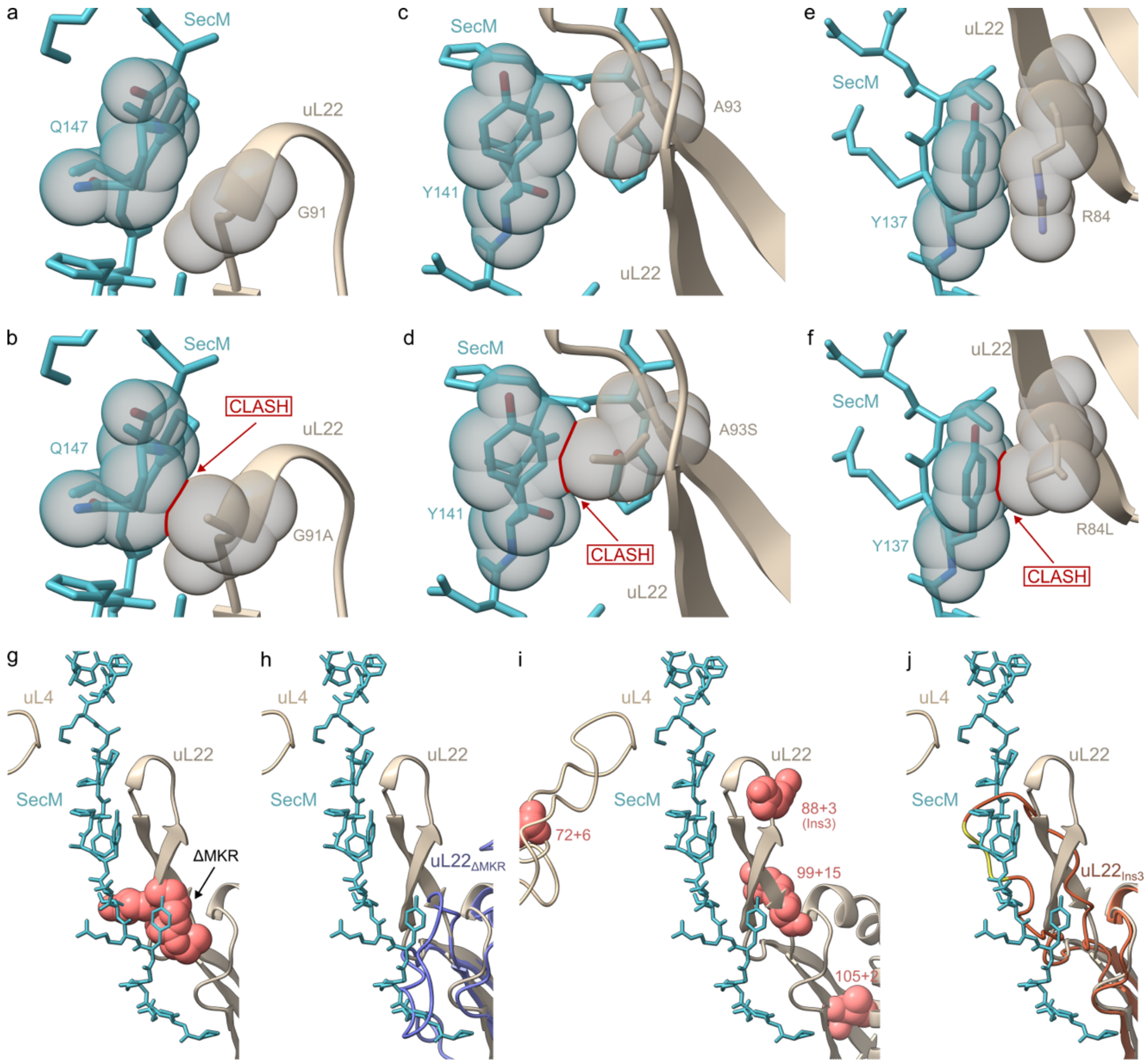

Supplementary Figure 5 Alterations in uL22 interfere with SecM-mediated stalling. (**a-b**) SecM (teal) together with native (a) and *in silico* mutated (b) G91A of uL22 (gold). (**c-d**) SecM (teal) together with native (c) and *in silico* mutated (d) A93S of uL22 (gold). (**e-f**) SecM (teal) together with native (e) and *in silico* mutated (f) R84L of uL22 (gold). (**g**) SecM (teal) together with uL4 (light gold) and uL22 (gold) and region of deletion in ΔMKR mutant of uL22 coloured in red spheres. (**h**) ΔMKR mutant of uL22 (purple) (PDB ID 1YJ9)[4] (aligned on the basis of the 23S rRNA) clashes with SecM nascent peptide (teal). (**i**) SecM (teal) together with uL4 (light gold) and uL22 (gold) and regions of insertion within uL22 coloured in red spheres. (**j**) Model with Ins3 mutant of uL22 (orange) (PDB ID 4WFN)[5] (aligned on the basis of the 23S rRNA) clashes with SecM nascent peptide (teal). Clashes between residues in sphere representation depicted as red line.

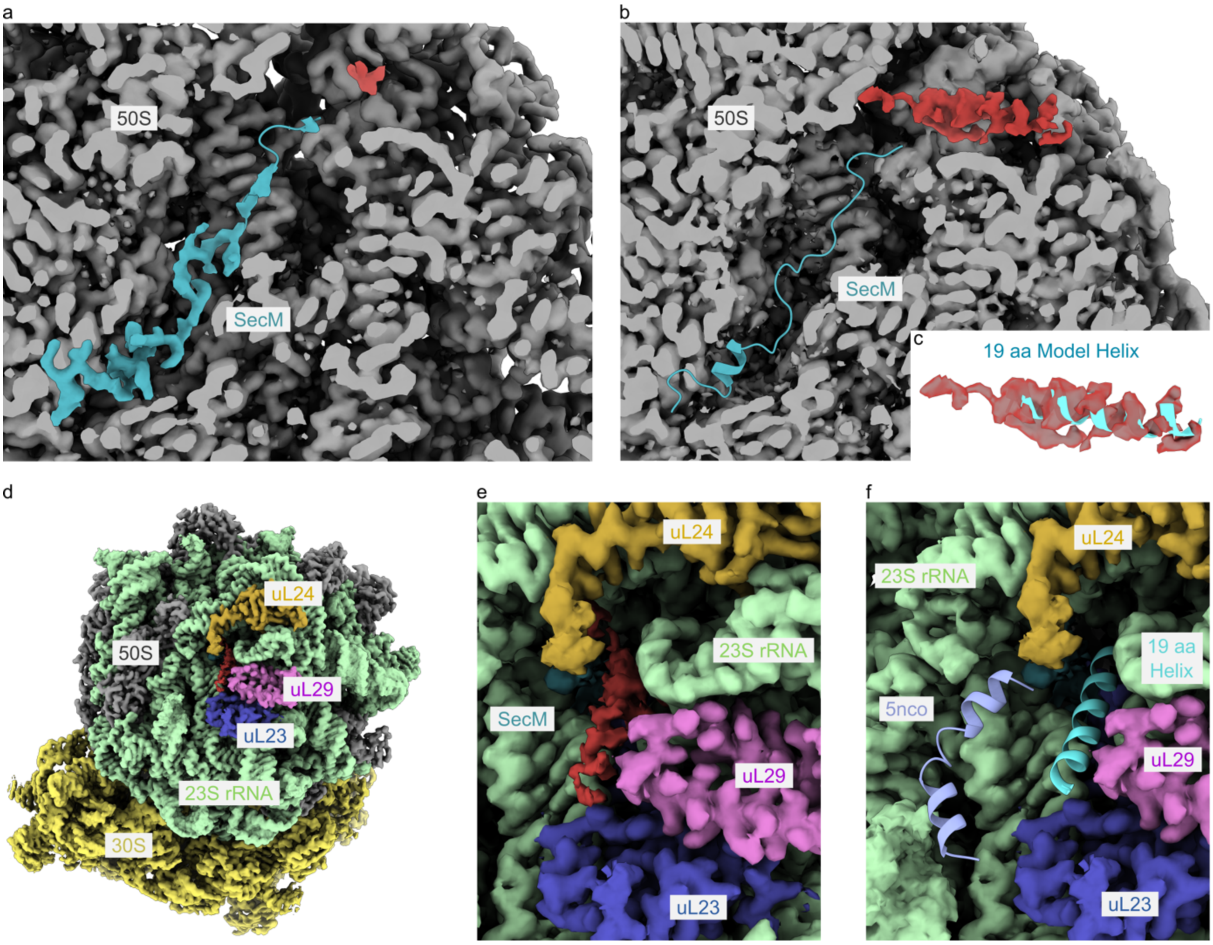

Supplementary Figure 6 Helical density at the exit of the NPET. (**a-b**) Transverse section of the 50S (grey) to reveal SecM (teal) and helical extra density at the exit of the NPET (red) (a) at same threshold as in figure 1d and (b) at lower threshold. (**c**) 19 amino acid α-helix (light blue) fit into extra density at the exit of the NPET. (**d-f**) Overview (d) of perspective for zoom representations (e-f) on helical density at the exit of the NPET. (**f**) Signal sequence (lavender) (PDB ID 5NCO)[6] (aligned on the basis of 23S rRNA) is localised at a different region at the exit of the NPET than the helical density.

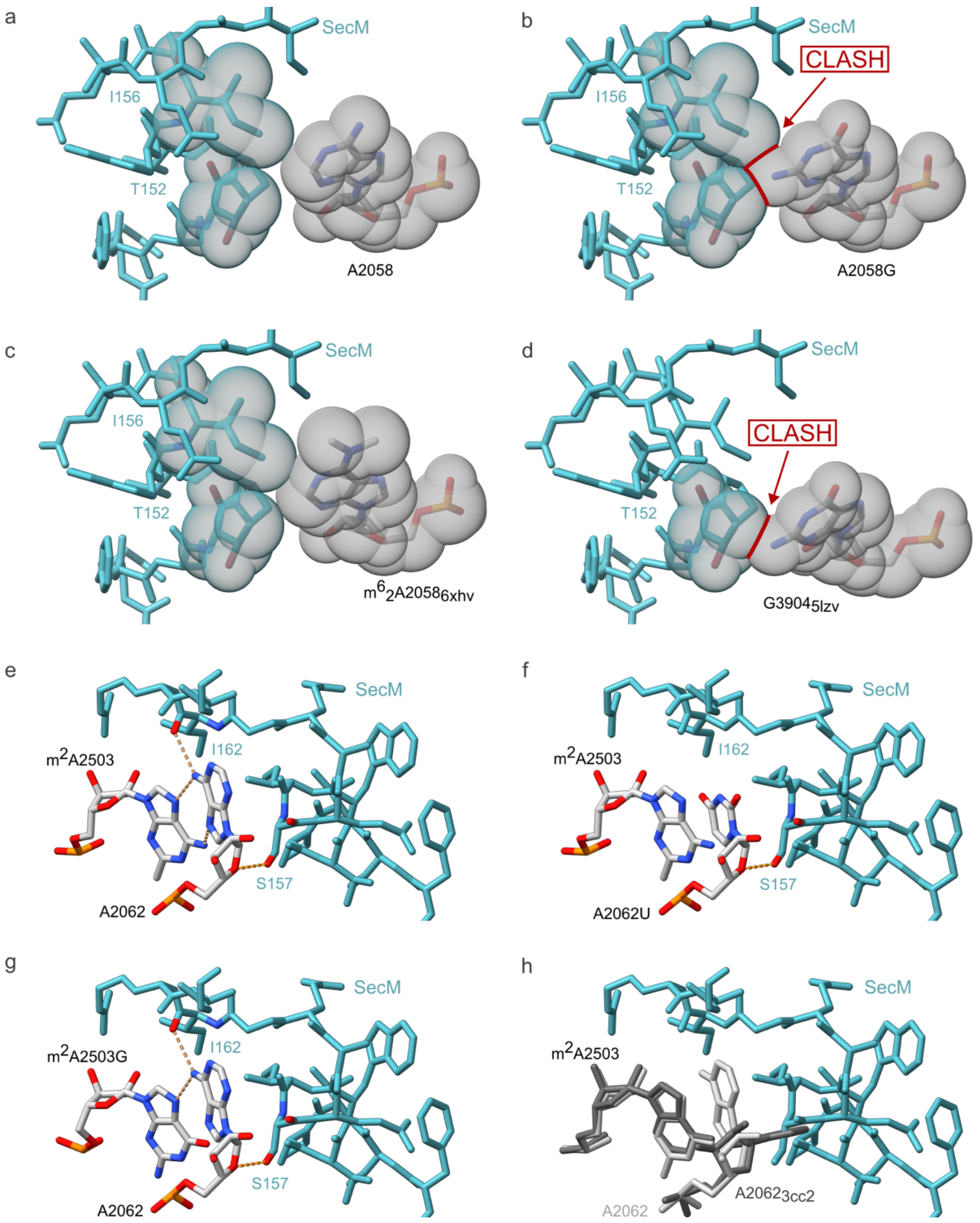

Supplementary Figure 7 Certain mutations of 23S rRNA nucleotides interfere with stalling of SecM. (**a**) Native conformation of A2058 (grey) together with SecM nascent peptide (teal). (**b**) *In silico* mutated A2058G together with SecM nascent peptide (teal). (**c**) $m^6{}_2$A2058 (grey) (PDB ID 6XHV)[7] (aligned on the basis of the 23S rRNA) together with SecM nascent peptide (teal). (**d**) Corresponding eukaryotic 28S rRNA nucleotide G3904 (grey) (PDB ID 5LZV)[8] (aligned on the basis of 23S and 28S rRNA) to *E. coli* A2058 together with SecM nascent peptide (teal). (**e**) Native conformation of m^2A2503 and A2062 (grey) together with SecM nascent peptide (teal). (**f**) Native m^2A2503 and *in silico* mutated A2062U (grey) together with SecM nascent peptide (teal). (**g**) *In silico* mutated m^2A2503G and native A2062 (grey) together with SecM nascent peptide (teal). (**h**) Overlay of m^2A2503 and A2062 (grey) from SecM structure and m^2A2503 and A2062 (dark grey) in non-bonded conformation (PDB ID 3CC2)[9] in which A2062 clashes with the SecM nascent peptide (teal). Potential hydrogen bonds shown as dashed orange lines and clashes between residues in sphere representation depicted as red line.

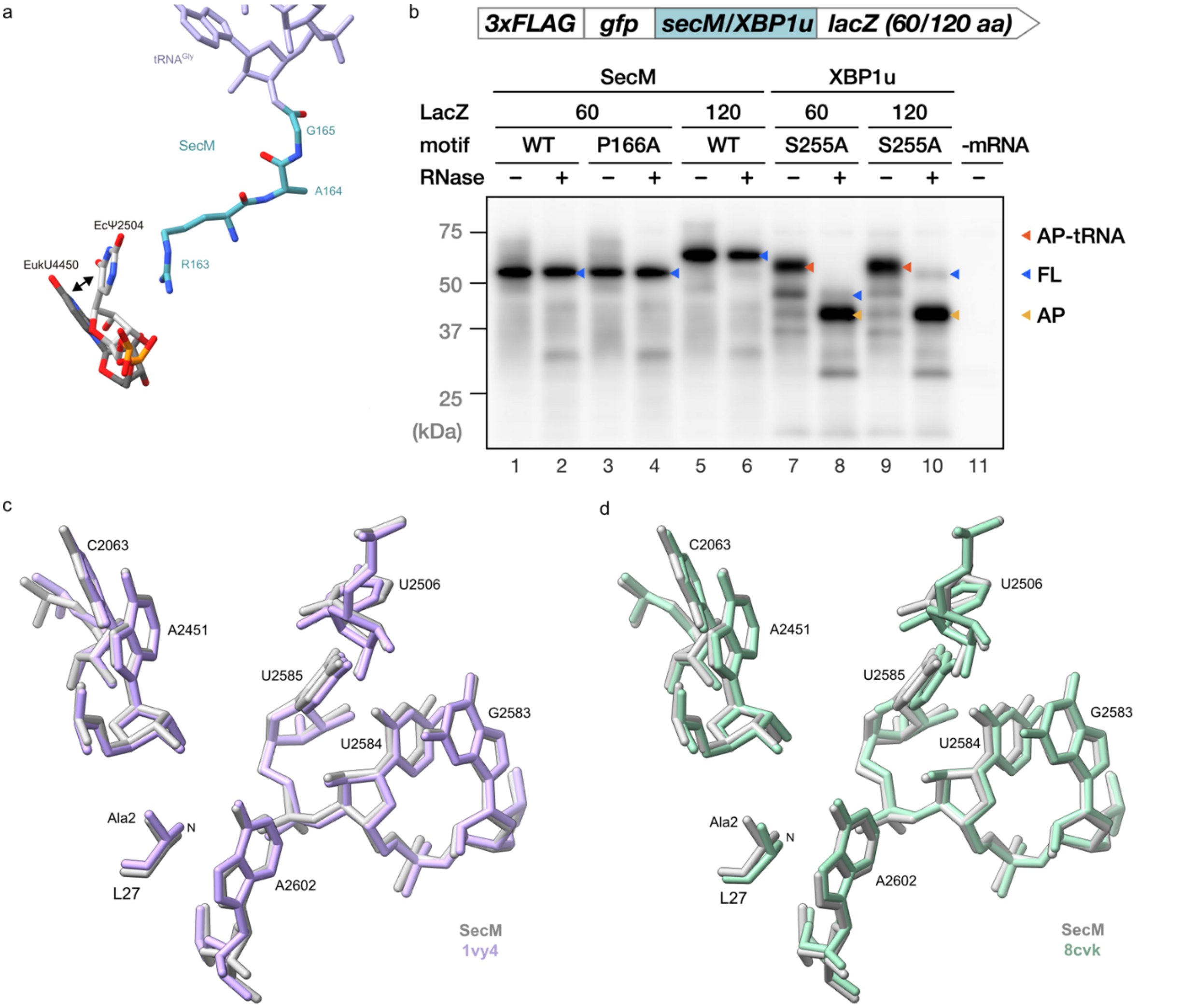

Supplementary Figure 8 SecM does not stall eukaryotic ribosome *in vitro* and conformation of ribosomal PTC components. (**a**) Corresponding eukaryotic 28S rRNA nucleotide U4450 (dark grey) (PDB: 5LZV) (aligned on the basis of 23S and 28S rRNA) to *E. coli* Ψ2504 (grey) together with $_{163}$RAG$_{165}$ from SecM nascent peptide (teal) attached to the P-tRNA (lavender). (**b**) A schematic representation of the *lacZ* reporter used for the *in vitro* assay (upper). The coding region for wild-type (WT) or arrest defective mutant derivatives (P166A) of SecM (38-170) or an arrest enhanced S255A derivative of XBP1u(186–261), was sandwich-fused between *gfp* and either *lacZ60* or *lacZ120*, which encodes the N-terminal 60 or 120 residues of LacZ, respectively. The *in vitro* translation in the rabbit reticulocyte lysate was carried out at 30°C for 20 mins. The translation products were analyzed by anti-FLAG immunoblotting. Full-length (FL: blue) and arrest species (AP) with (orange) or without tRNA moiety (yellow) are indicated with colored arrowheads. One of two biological replicates (n = 2) is shown, with source data and second replicate provided in Supplementary Figure 12. (**c-d**) Overlay of ribosomal components involved in the proton wire required for nucleophilic attack for peptide bond formation from SecM (grey) versus two pre-attack states (c) on the left (lavender) (PDB ID 1VY4)[10] and (d) on the right (green) (PDB ID 8CVK)[11]. Alignments are based on the 23S rRNA.

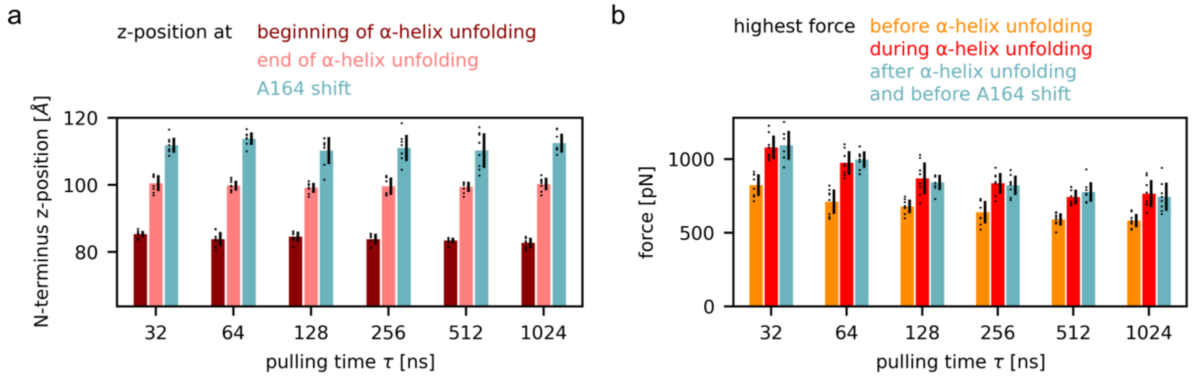

Supplementary Figure 9 Order of events and forces during pulling of the SecM N-terminus. (**a**) For each pulling time, the position of the N-terminal Pro132 is shown for three events: when the helix begins to unfold, when unfolding is completed, and when A164 shifts. Mean (bars) and standard deviations (black lines) obtained from 8 independent simulations (circles) for each pulling time. (**b**) For three intervals of the pulling simulations, before helix unfolding, during unfolding, and between completed unfolding and A164 shift, the highest forces were extracted from the simulations. For each pulling time and interval, mean (bars) and standard deviations (black lines) are shown for n = 8 independent simulations (circles). Source data be obtained from Zenodo (10.5281/zenodo.10492465).

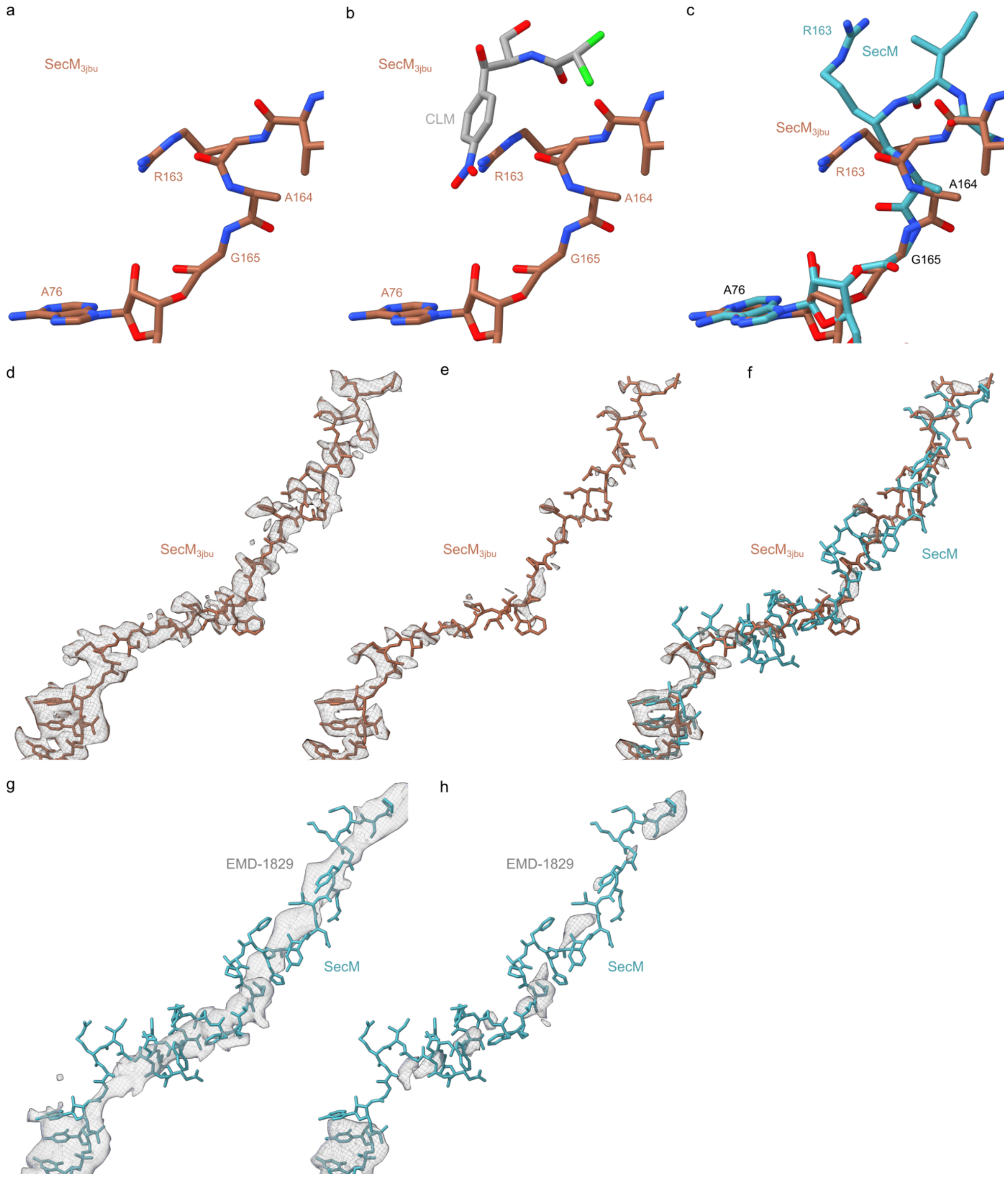

Supplementary Figure 10 Comparison of $SecM_{3JBU}$ and SecM. (**a**) Zoom on $SecM_{3JBU}$ $_{163}RAG_{165}$ (PDB ID 3JBU)[12] attached to the P-tRNA (brown) and (**b**) together with Chloramphenicol (grey) (PDB ID 7RQE)[13] (aligned on the basis of 23S rRNA). (**c**) Overlay of zoom on (a) $SecM_{3JBU}$ $_{163}RAG_{165}$ attached to the P-tRNA (brown) and SecM $_{163}RAG_{165}$ attached to the tRNA (teal) (aligned on the basis of 23S rRNA). (**d**) $SecM_{3JBU}$ nascent chain attached to the P-tRNA (brown) with extracted density in a radius of 2.5 Å at low threshold. (**e**) $SecM_{3JBU}$ nascent chain attached to the P-tRNA (brown) with extracted density in a radius of 2.5 Å at appropriate threshold for the tRNA and specifically the CCA-end. (**f**) Overlay of $SecM_{3JBU}$ nascent chain attached to the P-tRNA (brown) with extracted density in a radius of 2.5 Å at appropriate threshold for the tRNA and specifically the CCA-end and SecM attached to the P-tRNA (teal) (aligned on the basis of 23S rRNA). (**g-h**) Cryo-EM density for the SecM nascent chain (grey mesh) (EMD-1829)[14] shown at (**g**) low and (**h**) high threshold, and overlaid with the molecular model from SecM nascent chain (teal) determined in this study.

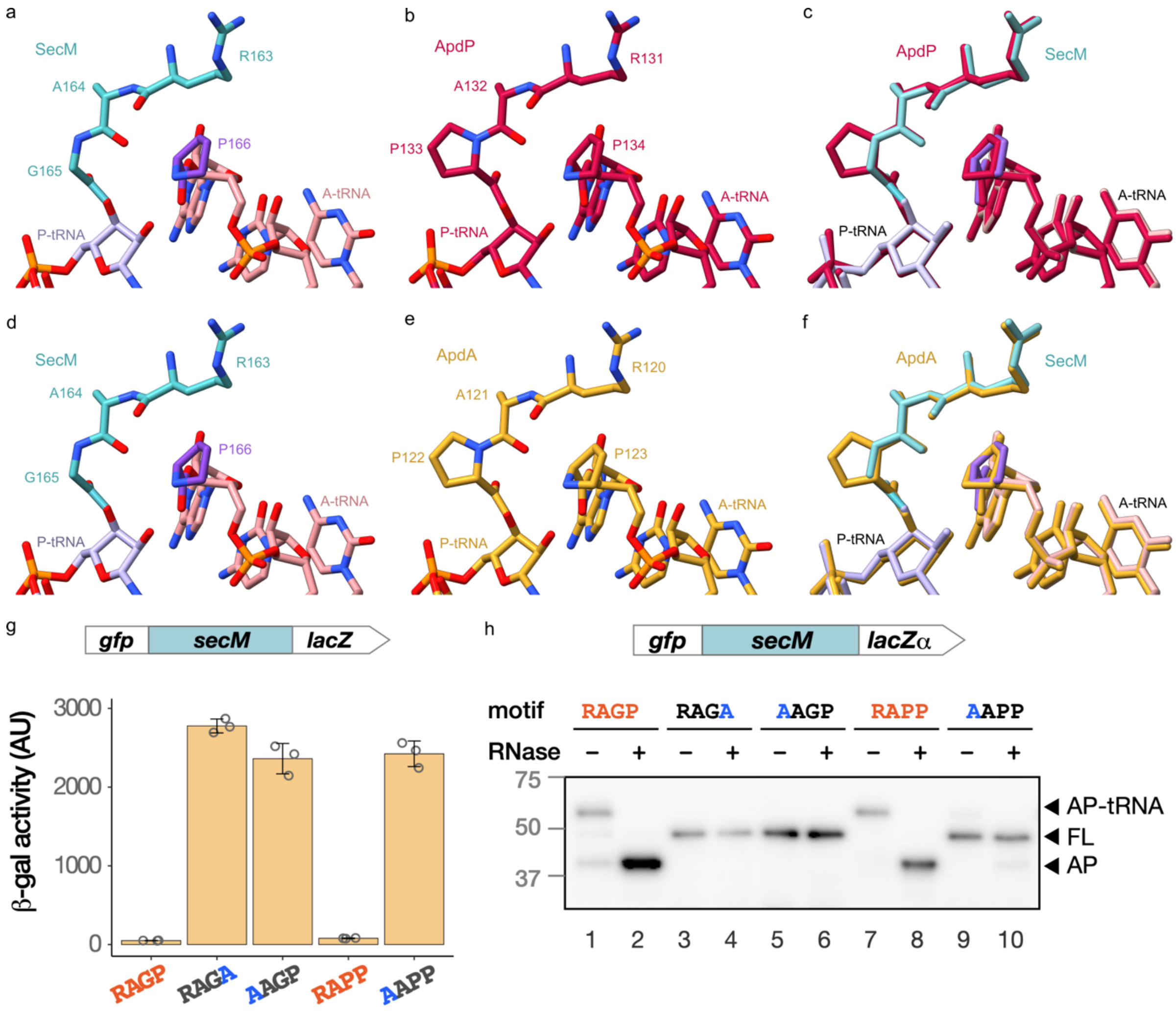

Supplementary Figure 11 Comparison of RAG/P and RAPP motifs of SecM and ApdP as well as ApdA arrest peptides and translation arrest assay of SecM mutant derivatives. (**a**) SecM $_{163}$RAG$_{165}$-tRNA (teal/lavender) in the P-site and Pro-tRNA (grape/salmon) in the A-site. (**b**) ApdP (PDB ID 8QBT)[15] $_{131}$RAP$_{133}$-tRNA in the P-site and Pro-tRNA in the A-site (red). (**c**) Overlay of (a) SecM $_{163}$RAG$_{165}$-tRNA (teal/lavender) in the P-site with Pro-tRNA (grape/salmon) in the A-site and (b) ApdP $_{131}$RAP$_{133}$-tRNA in the P-site with Pro-tRNA in the A-site (red) (aligned on the basis of the 23S rRNA). (**d**) SecM $_{163}$RAG$_{165}$-tRNA (teal/lavender) in the P-site and Pro-tRNA (grape/salmon) in the A-site. (**e**) ApdA (PDB ID 8QCQ)[15] $_{120}$RAP$_{122}$-tRNA in the P-site and Pro-tRNA in the A-site (yellow). (**f**) Overlay of (d) SecM $_{163}$RAG$_{165}$-tRNA (teal/lavender) in the P-site with Pro-tRNA (grape/salmon) in the A-site and (e) ApdA $_{120}$RAP$_{122}$-tRNA in the P-site with Pro-tRNA in the A-site (yellow) (aligned on the basis of the 23S rRNA). (**g**) A schematic representation of the *lacZ* reporter used for the *in vivo* assay (upper) and β-galactosidase activity (mean, n = 3) of *E. coli* cells harboring wildtype or mutant derivatives of *gfp-secM*$^{38\text{-}170}$*-lacZ* reporter (lower) in which the $_{163}$RAGP$_{166}$ sequence in the wild-type SecM was mutated to either RAGA, AAGP, RAPP, or AAPP. The error bars and dots represent standard deviations and individual data points from three biological replicates (n = 3), respectively. Source data are provided as a Source Data file. (**h**) Western blot analysis of *in vitro* translation products. The reporter genes (upper panel) harboring wild-type (WT) or mutant derivatives of the arrest motif indicated were translated in the PURE systems. The products were separated in neutral-pH gels and immunoblotted using anti-GFP. Before the separation, a portion of the samples were treated with RNase A (lanes indicated as +), to degrade the tRNA moiety. One of two biological replicates (n = 2) is shown, with source data and second replicate provided in Supplementary Figure 12.

a

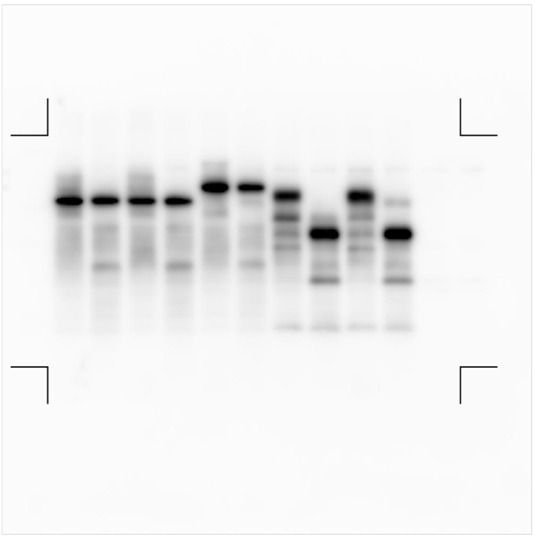

b

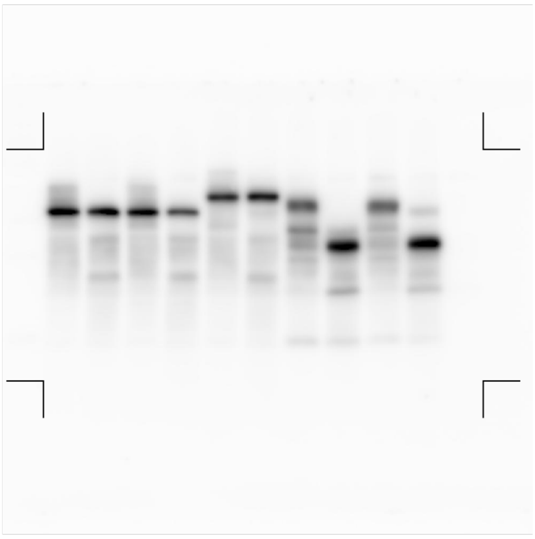

c

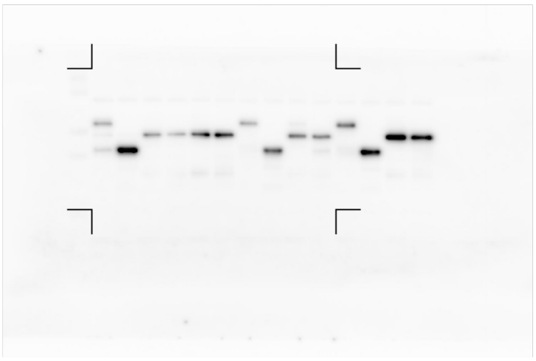

d

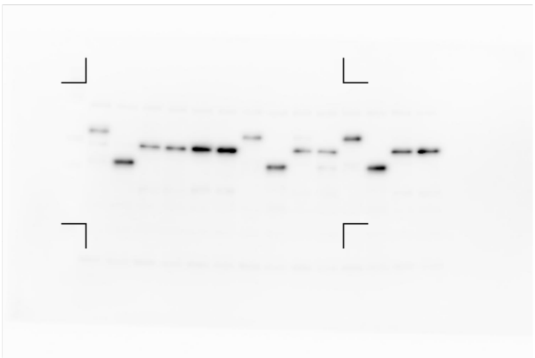

Supplementary Figure 12 Source Data for Supplementary Figure 8b and 11h. (**a**) Uncropped scans of the Western blots shown in Supplementary Figure 8b, including (**b**) additional scan of uncropped image from the second independent experiment. (**c**) Uncropped scans of the Western blots shown in Supplementary Figure 11h, including (**d**) additional scan of uncropped image from second independent experiment.

Supplementary Table 1: *E. coli* strains

strains	genotype	parent	plasmid	ref
KFE672	*gfp-secM(38-170)G165P-myc-lacZ-CTC*	JM109	pKIG1427	*
KFE675	*gfp-secM(38-170)R163A/G165P-myc-lacZ-CTC*	JM109	pKIG1433	*
KFE681	*gfp-secM(38-170)-myc-lacZ-CTC*	JM109	pCH2421	*
KFE682	*gfp-secM(38-170)P166A-myc-lacZ-CTC*	JM109	pCH2422	*
KFE683	*gfp-secM(38-170)R163A-myc-lacZ-CTC*	JM109	pCH2435	*

*This study

Supplementary Table 2: Plasmids

plasmid	gene	ref	PCR 1			PCR 2		
			fw primer 1	rv primer 1	template 1	fw primer 2	rv primer 2	template 2
pCH2139	gfp-secM(38-170)-flag-lacZ	(1)						
pCH2312	gfp-secM(38-170)-myc-lacZ	*	gfp238-fw	secM-myc27-rv	pCH2139	myc-lacZ7-fw	GFP238-rv	pSK69
pCH2313	gfp-secM(38-170)P166A-myc-lacZ	*	gfp238-fw	secMP166A-myc27-rv	pCH2139	myc-lacZ7-fw	GFP238-rv	pSK69
pCH2337	gfp-secM(38-170)R163A-myc-lacZ	*	secM-R163A-fw	ampR 121-128(Tm62)-antisense	pCH2312	ampR 121-128(Tm62)	secM-162-rv	pCH2312
pCH2421	gfp-secM(38-170)-myc-lacZ-CTC	*	myc-lacZ7-CTC-fw	myc27-rv	pCH2312	-	-	-
pCH2422	gfp-secM(38-170)P166A-myc-lacZ-CTC	*	myc-lacZ7-CTC-fw	myc27-rv	pCH2313	-	-	-
pCH2435	gfp-secM(38-170)R163A-myc-lacZ-CTC	*	myc-lacZ7-CTC-fw	myc27-rv	pCH2337	-	-	-
pKIG1354	CrPV-3xFLAG-gfp-XBP1u(186-261)S255A-myc-lacZ179	(2)						
pKIG1388	CrPV-3xFLAG-gfp-XBP1u(186-261)S255A-myc-lacZ179	(2)						
pKIG1427	gfp-secM(38-170)G165P-myc-lacZ-CTC	*	ampR 121-128(Tm62)	secM_Ile163 rv	pCH2421	secM_RAPP fw	ampR 121-128(Tm62).antisense	pCH2421
pKIG1433	gfp-secM(38-170)R163A/G165P-myc-lacZ-CTC	*	ampR 121-128(Tm62)	secM_Ile163 rv	pCH2421	secM_AAPP fw	ampR 121-128(Tm62).antisense	pCH2421
pKIG1453	CrPV-3xFLAG-gfp-secM(38-170)-myc-lacZ179	*	LacZ9 fw	GFP238-rv	pKIG1354	GFP231 fw	LacZ15 rv	pCH2421
pKIG1455	CrPV-3xFLAG-gfp-secM(38-170)P166A-myc-lacZ179	*	LacZ9 fw	GFP238-rv	pKIG1354	GFP231 fw	LacZ15 rv	pCH2422
pSK69	gfp-apdP(34-140)-lacZ	(1)						

(1) Reference [16]

(2) Reference [15]

* This study

Supplementary Table 3: Primers

Primer name	sequence (5' to 3')
ampR 121-128(Tm62)	GCAGTGCTGCCATAACCATGAGTG
ampR 121-128(Tm62).antisense	CACTCATGGTTATGGCAGCACTGC
GFP231 fw	CATGGCATGGATGAACTATACAAA
gfp238-fw	GGCATGGATGAACTATACAAA
GFP238-rv	TTTGTATAGTTCATCCATGCC
lacZ120-TAATAA-21rv	TGGTGCCGGAAACCAGGCAAATTATTACGGATTCTCCGTGGGAACAAA
LacZ15 rv	ACGACGTTGTAAAACGACGGC
lacZ60-TAATAA-21rv	TGGTGCCGGAAACCAGGCAAATTATTAGCGCCATTCGCCATTCAGGCT
LacZ9 fw	GCCGTCGTTTTACAACGTCGT
myc-lacZ7-CTC-fw	AAACTCATCTCAGAAGAGGATCTCTCACTCGCCGTCGTTTTACAA
myc-lacZ7-fw	AAACTCATCTCAGAAGAGGATCTGTCACTGGCCGTCGTTTTACAA
myc27-rv	ATCCTCTTCTGAGATGAGTTTTTGTTC
PT7 fw2	GGGCCTAATACGACTCACTA
PT7-RBSkf-GFP	TAACTTTAAGAAGGAGGGAGATATACCAATGACAATGTTTGTGGGATC
secM_AAPP fw	AGCCAGGCGCAAGGCATCGCTGCTCCTCCTCAACGCCTCACCGAA
secM_Ile163 rv	GATGCCTTGCGCCTGGCTTAT
secM_RAPP fw	AGCCAGGCGCAAGGCATCCGTGCTCCTCCTCAACGCCTCACCGAA
secM-162-rv	GATGCCTTGCGCCTGGCTTATCCAGAC
secM-myc27-rv	ATCCTCTTCTGAGATGAGTTTTTGTTCGGTGAGGCGTTGAGGGCCAGC
secM-R163A-fw	AGCCAGGCGCAAGGCATCGCTGCTGGCCCTCAACGC
secMP166A-myc27-rv	ATCCTCTTCTGAGATGAGTTTTTGTTCGGTGAGGCGTTGAGCGCCAGCACG
Universal-primer-77(PURE)	GAAATTAATACGACTCACTATAGGGAGACCACAACGGTTTCCCTCTAGAAATAATTTTGTTTAACTTTAAGAAGGAG

Supplementary Table 4: Preparation of template DNA for *in vitro* translation

gene	1st PCR			2nd PCR*		system
	fw primer 1	rv primer 1	template 1	fw primer 2	rv primer 2	
gfp-secM(38-170)-lacZa	PT7-RBSkf-GFP	lacZ60-TAATAA-21rv	pCH2421	Universal-primer-77(PURE)	lacZ60-TAATAA-21rv	PUREfrex
gfp-secM(38-170)P166A-lacZa	PT7-RBSkf-GFP	lacZ60-TAATAA-21rv	pCH2422	Universal-primer-77(PURE)	lacZ60-TAATAA-21rv	PUREfrex
gfp-secM(38-170)R163A-lacZa	PT7-RBSkf-GFP	lacZ60-TAATAA-21rv	pCH2435	Universal-primer-77(PURE)	lacZ60-TAATAA-21rv	PUREfrex
gfp-secM(38-170)G165P-lacZa	PT7-RBSkf-GFP	lacZ60-TAATAA-21rv	pKIG1427	Universal-primer-77(PURE)	lacZ60-TAATAA-21rv	PUREfrex
gfp-secM(38-170)R163A/G165P-lacZa	PT7-RBSkf-GFP	lacZ60-TAATAA-21rv	pKIG1433	Universal-primer-77(PURE)	lacZ60-TAATAA-21rv	PUREfrex
gfp-secM(38-170)-lacZ60	PT7 fw2	lacZ60-TAATAA-21rv	pKIG1453	-	-	RRL
gfp-secM(38-170)P166A-lacZ60	PT7 fw2	lacZ60-TAATAA-21rv	pKIG1455	-	-	RRL
gfp-secM(38-170)-lacZ120	PT7 fw2	lacZ120-TAATAA-21rv	pKIG1453	-	-	RRL
gfp-XBP1u(186-261)S255A-lacZ60	PT7 fw2	lacZ60-TAATAA-21rv	pKIG1388	-	-	RRL
gfp-XBP1u(186-261)S255A-lacZ120	PT7 fw2	lacZ120-TAATAA-21rv	pKIG1388	-	-	RRL

* The 1st PCR product was used as the template for the 2nd PCR

Supplementary References

1 Su, T. *et al.* The force-sensing peptide VemP employs extreme compaction and secondary structure formation to induce ribosomal stalling. *eLife* **6** (2017).

2 van der Stel, A. X. *et al.* Structural basis for the tryptophan sensitivity of TnaC-mediated ribosome stalling. *Nat Commun* **12**, 5340 (2021).

3 Matheisl, S., Berninghausen, O., Becker, T. & Beckmann, R. Structure of a human translation termination complex. *Nucleic acids Res* **43**, 8615-8626 (2015).

4 Tu, D., Blaha, G., Moore, P. & Steitz, T. Structures of MLSBK antibiotics bound to mutated large ribosomal subunits provide a structural explanation for resistance. *Cell* **121**, 257-270 (2005).

5 Wekselman, I. *et al.* The Ribosomal Protein uL22 Modulates the Shape of the Protein Exit Tunnel. *Structure* **25**, 1233-1241 e1233 (2017).

6 Jomaa, A. *et al.* Structure of the quaternary complex between SRP, SR, and translocon bound to the translating ribosome. *Nat Commun* **8**, 15470 (2017).

7 Svetlov, M. S. *et al.* Structure of Erm-modified 70S ribosome reveals the mechanism of macrolide resistance. *Nat Chem Biol* (2021).

8 Shao, S. *et al.* Decoding Mammalian Ribosome-mRNA States by Translational GTPase Complexes. *Cell* **167**, 1229-1240 e1215 (2016).

9 Blaha, G., Gurel, G., Schroeder, S. J., Moore, P. B. & Steitz, T. A. Mutations outside the anisomycin-binding site can make ribosomes drug-resistant. *J. Mol. Biol.* **379**, 505-519 (2008).

10 Polikanov, Y. S., Steitz, T. A. & Innis, C. A. A proton wire to couple aminoacyl-tRNA accommodation and peptide-bond formation on the ribosome. *Nat Struct Mol Biol* **21**, 787-793 (2014).

11 Syroegin, E. A., Aleksandrova, E. V. & Polikanov, Y. S. Insights into the ribosome function from the structures of non-arrested ribosome-nascent chain complexes. *Nat Chem* **15**, 143-153 (2023).

12 Zhang, J. *et al.* Mechanisms of ribosome stalling by SecM at multiple elongation steps. *eLife* **4** (2015).

13 Syroegin, E. A. *et al.* Structural basis for the context-specific action of the classic peptidyl transferase inhibitor chloramphenicol. *Nat Struct Mol Biol* **29**, 152-161 (2022).

14 Bhushan, S. *et al.* SecM-stalled ribosomes adopt an altered geometry at the peptidyltransferase center. *PLoS Biol.* **19**, e1000581 (2011).

15 Morici, M. *et al.* RAPP-containing arrest peptides induce translational stalling by short circuiting the ribosomal peptidyltransferase activity *Nat. Commun.* (2024).

16 Fujiwara, K., Tsuji, N., Yoshida, M., Takada, H. & Chiba, S. Patchy and widespread distribution of bacterial translation arrest peptides associated with the protein localization machinery. *BioRxiv preprint* **2023.09.02.556018** (2023).
