# Supplementary material for: The SecM arrest peptide traps a pre-peptide bond formation state of the ribosome
Source: Nat Commun. 2024 Mar 19;15:2431. doi: 10.1038/s41467-024-46762-2 (PMC10951299; doi:10.1038/s41467-024-46762-2)
Supplement: Supplementary file 3 — Description of Additional Supplementary Files [file 41467_2024_46762_MOESM3_ESM.pdf]

Description of Additional Supplementary Files

File name: Supplementary Movie 1
Description: Cryo-EM density and model for the VemP nascent chain and Pro166 attached to its tRNAs. Video showing the quality of the cryo-EM density (black mesh) with additional density at lower threshold for N-terminal part of nascent chain (grey mesh) and fit of the molecular model of the nascent chain (teal) attached to the P-tRNA (lavender) and Pro166 (grape) attached to the A-tRNA (salmon). Second part of the video shows a zoom of the arrest motif including the α-helix in the nascent chain.

File name: Supplementary Movie 2
Description: Stalling release by pulling on N-terminus of SecM. Trajectory obtained from a 1024-ns MD simulation with a moving harmonic potential acting on the N-terminal residue. The events of helix unfolding and Ala164 shift are indicated. Colors and representations as described in Fig. 6b.
